# Screen-Printed Electrodes (SPE) for In Vitro Diagnostic Purpose

**DOI:** 10.3390/diagnostics10080517

**Published:** 2020-07-26

**Authors:** Nicolae-Bogdan Mincu, Veronica Lazar, Dana Stan, Carmen Marinela Mihailescu, Rodica Iosub, Andreea Lorena Mateescu

**Affiliations:** 1 Department of Botany and Microbiology, Faculty of Biology, University of Bucharest, 060101 Bucharest, Romania; nicolae.mincu@drd.unibuc.ro (N.-B.M.); veronica.lazar2009@gmail.com (V.L.); 2 DDS Diagnostic, 032032 Bucharest, Romania; dana_stan@ddsdiagnostic.ro (D.S.); carmen_mihail28@yahoo.com (C.M.M.); iosub.rodica@yahoo.com (R.I.); 3 National Institute for Research and Development in Microtechnologies (IMT), 077190 Bucharest, Romania

**Keywords:** screen-printed electrodes, biosensors, diagnosis

## Abstract

Due to rapidly spreading infectious diseases and the high incidence of other diseases such as cancer or metabolic syndrome, there is a continuous need for the development of rapid and accurate diagnosis methods. Screen-printed electrodes-based biosensors have been reported to offer reliable results, with high sensitivity and selectivity and, in some cases, low detection limits. There are a series of materials (carbon, gold, platinum, etc.) used for the manufacturing of working electrodes. Each version comes with advantages, as well as challenges for their functionalization. Thus, the aim is to review the most promising biosensors developed using screen-printed electrodes for the detection/quantification of proteins, biomarkers, or pathogenic microorganisms.

## 1. Introduction

Lately, there has been a high demand for sensitive, specific, rapid, and precise analysis methods using screen-printed electrodes. Screen-printed and interdigitated electrodes allow a large number of experiments to be undertaken, with low volumes of reagents and samples and without electrode pretreatment or maintenance.

Screen-printed electrodes (SPE) are usually used for measurements carried out for research in areas such as medicine, pharmacy, food industry, agriculture, environment or national security.

The screen-printed electrodes can be selected according to the material from which the working electrode is made, for example carbon, gold, platinum, or other metals, depending on the ongoing study. Due to the high reproducibility between electrodes, these sensors are suitable for a wide range of applications: clinical sensing platforms development, environmental, and food industry analysis. Moreover, sensor arrays allow the determination of multiple substances in parallel.

The purpose of the review is to highlight the latest results in the development of screen-printed electrodes, including microelectrodes and modified electrodes, which has recently led to new possibilities in the detection, identification, and quantification of biomolecules, drug, antigens, pathogenic microorganisms, and enzymes.

Lately, research for the development of biosensors has exploded, becoming a field of research for each type of biosensor, i.e., DNA (Deoxyribonucleic acid)-based sensors (genosensors), aptasensors, immunosensors, and enzymatic biosensors. The focus of this study is to investigate different functionalization and immobilization methods to capture molecules (e.g., proteins, antibodies, oligonucleotides, etc.). This investigation is the basis of expanding the development of new electrochemical sensors.

This review describes the basic fabrication principles and configuration design of screen-printed electrodes and electrochemical applications for the determination and identification of drugs, pathogenic microorganisms, virus and protein biomarkers for clinical analysis purposes, and avoidance of human health problems. To our knowledge, there is currently no published review paper that focuses on the use of SPE to detect bacterial pathogens, virus, illicit drugs, and diseases such as cancer and the metabolic syndrome. Moreover, the presently available methods for the detection and quantification of metabolic syndrome (MS) specific markers (C-reactive protein) by modified screen-printed electrodes has not been summarized in any other paper.

This review includes an introduction, an overview of screen-printed electrodes, a detailed summary of the most recent functionalization methods of the working electrode, and the immobilization methods for aptamers, proteins, antibodies, cancer biomarkers, amino acids, microorganisms, and illicit drugs. Further, this review contains information about the methods of choice for electrochemical analysis, such as cyclic voltammetry or impedance measurements carried out using a potentiostat/galvanostat [1].

Studies using screen-printed electrodes and electrochemical analysis have demonstrated that the working electrode (the material from which it was made) exhibits a major role in the modulation of the electrochemical response and the functionalization process by proper immobilization of the element responsible to detect the targeted analyte.

## 2. The Basic Fabrication Principles, the Configuration Designs of the Screen-Printed Electrodes

Recently published articles have revealed that screen-printed electrodes with excellent performance are mainly based on imports from America (Gamry Instruments) or from (Ω Metrohm DropSens) headquartered in Asturias, although there are some manufacturers in Europe and China, which are also specialized in the design of instruments for electrochemistry research. Marketed screen-printed electrodes are made on substrates such as ceramic, glass, or transparent flexible plastic. The Gamry Instruments site and Ω Metrohm DropSens site offer a wide range of screen-printed electrodes modules and corresponding potentiostats/galvanostats (some of them portable). Measurements made using the main electrochemical methods seem to maintain the precision of the larger instruments and are also easy to use with a computer interface.

**Figure 1:**
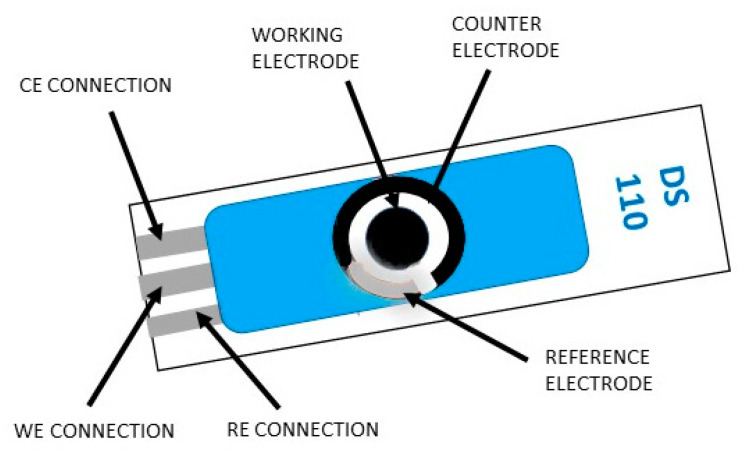
Electrode module used for electrochemical analysis.

A screen-printed module with three electrodes contains a working electrode, auxiliary electrode (counter electrode), and reference electrode, while the one with four electrodes contains a working electrode, working sense, auxiliary electrode (counter electrode), and reference electrode. The last configuration is usually used to measure the effect of an applied current on a solution or some barrier in that solution. There is also the possibility of producing personalized electrodes (different designs and materials) according to the specifications of the researcher. The screen-printed electrodes available upon request are made from carbon, gold, platinum, silver, or carbon nanotube inks. Currently, many studies are focusing on the development of screen-printed electrodes modified with nanomaterial such as graphene, carbon, metal, or quantum dots. An increased tendency to use fabricated SPEs for electrochemical analysis has been observed in various domains, such as environmental, clinical, or agro-food areas [2]. The screen-printed electrodes are single use devices, inexpensive, specially designed to work with sample microvolumes, and they can be subjected to electrochemical analysis by various methods, such as cyclic voltammetry (CV), differential pulse voltammetry (DPV), square wave voltammetry (SWV), chronoamperometry (CA), and chronopotentiometry (CP). These methods are used to avoid problems that arise when using traditional electrodes. They are used successfully in the most varied fields that involve electrochemical analysis methods, such as quality control, research, detection of a wide range of analytes (i.e., antigens [3], enzymes [4,5], and heavy metal ions [6] with a high degree of efficiency), and sensitivity [1]. As an example, Figure 1 shows a screen-printed electrode module that can be attached to a potentiostat used in the electrochemical analysis and taken from Ω Metrohm DropSens Spain site.

An extremely important step in establishing the performance of the carbon screen-printed electrode in the electrochemical analyzes is the establishment of the hydrophilicity of the working electrode surface.

Studies have shown that alkaline treatment with sodium hydroxide on the working electrode of the carbon screen-printed electrodes significantly increases the electron transfer by increasing the sensitivity and thus a visible improvement of their electrochemical performances occurs [7,8].

## 3. Screen-Printed Electrodes to detect Pathogens

Some scientists focused on the detection of pathogenic food born microorganisms, while others target the etiological agents for different infectious diseases with great importance for medical microbiology sector.

### 3.1. Detection and Quantification of Food-Borne Pathogens Using Screen-Printed Electrodes

In a study carried out by Viswanathan et al. [9], they were able to develop an immunosensor using screen-printed electrodes by immobilizing a mixture of anti-*Escherichia coli*, anti-*Campylobacter* and anti-*Salmonella* antibodies. Antibodies were bound to a multiwall carbon nanotube-polyallylamine modified screen-printed electrode with a ratio of 1:1:1. They reported a detection limit (LOD) for *Salmonella sp*. and *Campylobacter sp.* of 400 cells/mL and 800 cells/mL for *E. coli* and also concluded that the precision and sensibility of the multiplex made this method feasible for the determination of bacteria in milk samples [9]. In another study, also targeting the rapid detection of *Salmonella sp.* from food, the researchers developed an immunosensor using screen-printed gold working electrodes and silver pseudo-reference electrode. They tied monoclonal antibodies to the surface of the working electrode using physical and covalent immobilization by amine coupling of carboxymethyl dextran. Their results showed good selectivity for other bacterial strains, but cross-reactivity with other members of the *Enterobacteriaceae* family was still possible. The authors suggested that further testing was necessary [10].

Other scientists were able to identify the targeted pathogen not only at a genus level but also at the species level. Such an example is a recent study carried out by Fei et al. [11], where they constructed a sandwich like electrochemical immunoassay to detect *Salmonella pullorum* in food samples. Basically, they used immunomagnetic beads for capturing and enrichment of *S. pullorum* from the samples and reduced graphene oxide (rGO) with gold nanoparticles (AuNPs) as the electrochemical label. The immunobeads were developed by conjugation of the first antibody to the silica coated magnetic beads and the rGO/AuNPs were obtained by co-reduction of the chloroauric acid and graphene oxide and bound to the second antibody. This complex structure was then immobilized on the working electrode [11].

In another study, self-assembled monolayers (SAMs) and gold screen-printed electrodes (AuSPEs) were used to develop immunosensors for the detection and quantification of *E. coli*. They constructed two configurations, one was the covalent immobilization of the antibody using cross-linker 3,3′-dithiobis (sulfosuccinimidylpropionate) and the other was obtained using thiolated antibodies immobilized to the electrode surface. They were able to detect and accurately quantify up to 3.3 CFU (colony forming units)/mL [12].

One of the issues when using antibodies-based biosensors to detect pathogens is specificity, because usually they are not the only contaminants and other similar microorganisms could trigger false results. Garcíaa et al. [13] were able to avoid this issue using a thin-film gold-electrode. They immobilized a thiolated sequence, which was complementary to the target and the hybridization with the target was signaled by the ruthenium complex, and hence used as an electrochemical indicator to show that hybridization took place [13].

**Figure 2:**
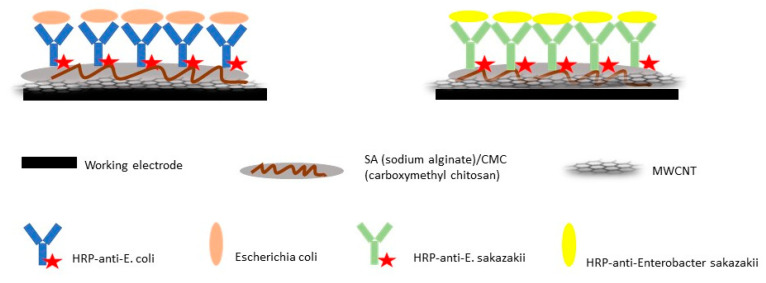
Structural illustration of the immuno-electrodes for the determination of *E. coli* O157:H7 and *E. sakazakii* [14].

Using carbon screen-printed electrodes, researchers were able to develop an electrochemical immunosensor to detect *E. coli* O157:H7 and *Enterobacter sakazakii* [14]. The multi-walled carbon nanotubes (MWCNT) sodium alginate and carboxymethyl chitosan composite films were coated on all the working electrodes in order to make them more sensitive (Figure 2). Antibodies for *E. coli* O157:H7 and *E. sakazakii* were labeled with horseradish peroxidases (HRP) and were immobilized on different screen-printed electrodes. The immobilization of the HRP antibodies was investigated using atomic force microscopy and cyclic voltammetry [14].

The detection of pathogens such as *Listeria monocytogenes* can take up to one week through the currently standardized methods and during this time a small issue can turn into an epidemic, thus faster and more reliable methods have been researched. Screen-printed electrodes could represent an excellent solution for this issue. In a study carried out by Davis et al. [15], gold modified carbon screen-printed electrodes were used to immobilize anti-*L. monocytogenes* antibodies. Using this model, they were able to detect densities as low as 2 CFU/g in approximately 1 h [15].

Other researchers focused on the immobilization of single stranded specific DNA. One study used gold screen-printed electrodes (SPE) to immobilize a thiolated 25 base single stranded probe of the lac Z gene (encodes for β-galactosidase) specific to members of the *Enterobacteriaceae* family [16]. Two configurations were designed with thiolated and biotinylated probes, using methylene blue as indicator or enzymatic signal amplification using peroxidase and TTF (tetrathiafulvalene). Their results showed good repeatability and reproducibility, and they suggested mass production for this type of genosensors for the rapid and reliable identification of *Enterobacteriaceae* in food.

In a recent study, researchers developed an impedance biosensor using immunomagnetic nanoparticles, screen-printed electrodes, and urease for the amplification of the electrochemical signal, to detect *L. monocytogenes*. A complex called MNP (magnetic nanoparticles coated with the antibodies)–Listeria–GNP (gold nanoparticles coated with antibodies and urease) was formed and resuspended in urea in order to induce its hydrolysis into ammonium ions and carbonate ions, which were measured by the electrode [17].

Gomez et al. [18] developed an amperometric immuno-sensing design to detect *Staphylococcus aureus*, based on the immobilization of Rb IgG (Rabbit IgG) on gold plated electrodes using a cross-linker 3,3-dithiodipropionic acid di(N-succinimidyl ester) (DTSP). They reported good analytical performance for their models, especially the antiRbIgG-HRP, cell wall lysis by ultrasonication and Au/SPEs, through which they were able to detect 3.7 × 10^2^ CFU mL [18].

Ward et al. [19] were able to construct a low-cost screen-printed electrode to detect *S. aureus.* In this study, electrodes were screen-printed onto a polyethylene terephthalate (PET) substrate using a solvent cure carbon ink. They conducted measurements using electrochemical impedance spectroscopy (EIS) and obtained specific impedance signatures for *S. aureus* in LB (Lysogeny broth), FBS (fetal bovine serum) and 0.9% NaCl media and concluded that through this method screen- printed electrodes can be used without the need of any surface recognition elements, thus making it a cheaper model compared to previously described ones [19].

Researchers also paid attention to less frequent food pathogens such as *Vibrio parahaemolyticus*, an immunosensor for rapid detection of this pathogen proposed by Zhao et al. [20]. The authors developed a model using screen-printed electrodes coated with agarose/Nano-Au membrane and specific antibodies that were labeled with horseradish peroxidase (HRP). The detection limit for *V. parahaemolyticus* was 7.374 × 10^4^ CFU/mL and the analysis had good specificity and selectivity and excellent accuracy [20].

### 3.2. Detection and Quantification of Infectious Diseases Etiological Agents Using Screen-Printed Electrodes

According to the data provided by the Joint United Nations Program on HIV and AIDS (UNAIDS) [21], an estimated 37.9 million people were living with HIV (human immunodeficiency virus) at the end of 2018. Thus, a rapid method of detecting the virus before it can spread further is necessary. A study addressing this need was carried out by Lam Dai Tran et al. [22], who constructed a CS/Fe_3_O_4_ nanocomposite platform to detect HIV-1 using an electrochemical method. This platform had a low detection limit (up to 50 pM) and good reproducibility. Another study developed a rapid and cost-efficient method to detect HIV and an influenza virus, subtype H5N1. Two models were used, one based on paramagnetic nanoparticles covered with streptavidin and specific oligonucleotide sequences labeled with biotin and the other with carbon nanotubes-based screen-printed electrodes, and a micro flow instrument to detect viral nucleic acids. They concluded that both methods used in the study are suitable for an easy and rapid detection of viral nucleic acid [23].

Electrochemical genosensors are evolving at a fast pace due to their extended use for various applications in different domains [24]. SPE-based genosensors have already been reported by many researchers. For instance, Martinez-Paredes et al. [25] developed a hybridization-based genosensor on gold modified carbon screen-printed electrodes to detect SARS (severe acute respiratory syndrome) virus. They reported a detection limit of 2.5 pmol/L and a sensitivity 1.76 mA/pmol L^−1^. According to the data provided by the World Health Organization (WHO), 2003 SARS epidemy affected 26 countries and around 8000 people, and still there is no developed vaccine, which means that the threat of another epidemy is still very real [26]. A rapid and specific method for the identification of the virus in its early stages of infection would help to prevent an outbreak and SPE-based genosensors could represent our chance to do so. Wang et al. [27] also developed an electrochemical DNA-based sensor to detect short specific sequences of the human immunodeficiency virus type 1 (HIV-1).

Another important public health concern is viral hepatitis, especially B and C types. The development of rapid and cheap testing methods would allow a more frequent screening of the population/patients and better control when it comes to virus dissemination. Immunosensors to detect hepatitis B virus had already been reported [28]. They used magnetic beads and gold nanoparticles for the electrochemical detection of human IgG antibodies anti-hepatitis B surface antigen (HBsAg).

A recent study carried out by Valipour and Roushani describes a sandwich type immunosensor used to detect a HCV (hepatitis C virus) core antigen via screen-printed carbon electrodes, modified with a Nafion/TiO_2_ nanocomposite and loaded with a second antibody to entrap celestine blue (CB). The antigen and complex CB/Ab2/Nafion/TiO_2_ were conjugated to a sandwich type structure. Differential pulse voltammetry was used to detect the signal and the intensity of the signal was related to the concentration of the targeted antigen. Tests carried out on human serum samples revealed a high recovery rate (97% to 100.5%) and good precision [29].

Scientists also developed a model using screen-printed electrodes modified with thiophene to detect Dengue virus, targeting the marker NS1 protein [30]. The thiophene SPE was covered with gold nanoparticles conjugated to protein A and the anti-NS1 antibodies were immobilized to the surface using their Fc regions. They used cyclic voltammetry to detect amperometric responses of the targeted protein and used ferrocyanide/ferricyanide as redox probe. The detection limit using the modified SPE model they developed was very low (0.015 µg/mL^−1^), which means that early detection of the virus and epidemic control can be achieved [30].

Other scientists focused on finding rapid and accurate methods using screen-printed electrodes to detect *Vibrio cholerae* (the etiological agent of the well-known gastrointestinal disease, cholerae). As reported by other authors, cholerae is still a threat, especially in economically weak countries and poor hygiene conditions [31]. Rao et al. [32] developed a *V. cholerae* detection method using SPE and specific antibodies (harvested from rabbits and mice). They used an amperometric detection method and also standard enzyme-linked immunosorbent assay (ELISA) and concluded that the first method is faster with increased sensitivity.

Other important pathogens related to human illness are from the filum *Amoebozoa* such as *Acanthamoeba*, *Entamoeba*, etc. The prevalence of infections with these pathogens is also higher in developing countries due to poor hygiene conditions and the diagnosis is often unreliable due to costs and logistics [33]. A very promising and cost-efficient method to detect *Entamoeba histolytica* was proposed by Grewal et al. [34]. The method they proposed is based on the detection of cyst specific antigens using screen-printed electrodes and yeast bound single chain fragment variable antibody (yeast-scFv) [34]. 

Group A streptococci are also a group of microorganisms often related to invasive and noninvasive human infections. Because there is a high incidence of infections caused by GAS strains the development of rapid, accurate and cost-efficient methods for detection. Ahmed et al. [35] were able to specifically detect *Streptococcus pyogenes* in human saliva using commercial gold screen- printed electrodes and polytyramine (Ptyr). They used biotin-tagged specific antibodies conjugated to Ptyr amine group through the biotin-neutravidin coupling. The selectivity of this impedimetric immunosensor was high and the linear response was 100 cells/10 μL to 10^5^ cells/10 μL in cumulative incubation and 100 cells/10 μL to 10^4^ cells/10 μL in single shot incubation [35].

A more detailed presentation of the functionalization patterns of different types of SPE and the immobilization methods for the capture probes, to detect pathogens is shown in Table 1.

## 4. Illicit Drug Detection Using Screen-Printed Electrodes

According to the data provided by the UNODC (United Nations Office on drugs and crime), around 35 million people are estimated to suffer from drug use disorder [36]. It is a well-known fact that drug dependence is a developmental disorder that begins with experimental use in adolescence [37]. Drug use disorder is characterized by compulsive drug intake and loss of control and social and occupational impairment [38]. Reports also provided by UNODC show alarming quantities of drugs have been distributed around the world, such as heroin (430–450 tons annually) and cocaine (470 tons annually) [36]. Without proper control measures this will only lead to an increased number of people suffering from drug disorder, especially the young ones. These data suggest there is a great need to develop rapid, low cost, and reliable methods not only for the population screening for drug consumption but also for a fast tracking of drug distribution, before they reach the consumer and create irreversible damage. We will now look at some promising methods to detect traces of various illicit drugs on surfaces/materials or in biological samples.

**Figure 3:**
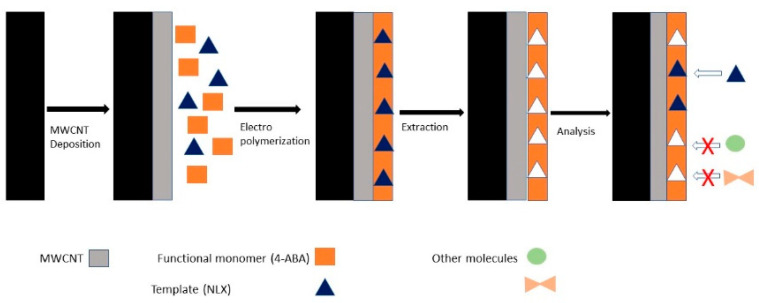
Schematic illustration of the preparation of the molecularly imprinted polymer (MIP)/multi-wall carbon nanotubes (MWCNT)/screen-printed carbon electrode (SPCE) [39].

In a recent study, researchers developed an electrochemical sensor to detect naloxone (morphine derivate) using commercial available screen-printed electrodes modified through the MIP technique (molecularly imprinted polymer) and multi-wall carbon nanotubes (MWCNT) by electropolymerization using cyclic voltammetry [39]. The structure of the sensor is described in Figure 3.

Beitollahi et al. [40] used magnetic core shell manganese ferrite nanoparticles to modify carbon screen-printed electrodes for obtaining a sensor for morphine measurement. The analysis of the electrochemical behavior of morphine was carried out using cyclic voltammetry. An interesting fact is that they studied the electrochemical behavior of morphine at different pH (3.0–9.0) and found that electro-oxidation of morphine at the surface of the sensor was optimum at pH 7.0 [40].

Cannabis is one of the most widely used drugs among European citizens, with usage percentages as high as 11% of the population (France and Spain) having smoked marijuana over a period of 12 months [36]. Cannabis consumption causes sleepiness, euphoria, modifications in the auditory and visual perception, and decreased psychomotor functions, which can lead to serious accidents and in some cases death. A study from 2016 focused on the development of a rapid and efficient method for the screening of drivers for cannabis onsite. Wanklyn et al. [41] created a disposable sensor using screen-printed electrodes to detect delta-9-tetrahydrocannabinol (Δ9-THC) in saliva samples. The screen-printed electrodes were coated with N-(4-amino-3-methoxyphenyl-methanesulfonamide) (OX0245). After OX0245 oxidation to the diimine, it reacts with Δ9-THC forming an adduct with two resonance structures, which can also be reduced by the diimine of resonance IV, resulting in an increase of the reduction current at the diimine reduction potential [41]. They tested this model on saliva samples from cannabis smokers and the detection limit was 25–50 ng/mL Δ9-THC [41].

Screen-printed sensors were also used to detect methamphetamine. The researchers used a screen-printed carbon electrode and a mediator (N,N′-(1,4-phenylene)-dibenzenesulfonamide) that once oxidized will react with methamphetamine (MAMP) and form an adduct that can be subjected to electrochemical reduction [42]. Using a double square wave voltammetry technique, they were able to detect concentrations of MAMP as low as 400 ng/mL in 55 s. They concluded that although the response time of the sensor was very good, the detection limit was higher than what currently used methods can ensure and further work is needed for the improvement of the method. In another study they constructed an amperometric immunosensor for the specific detection of 3,4-methylenedioxyamphetamine and its derivates. The immunosensor consisted of carbon screen- printed electrodes, which were incubated in the presence of the specific antibody solution, in humid conditions, in order to induce passive absorption [43]. The immunosensor showed high specificity towards the targeted compounds even when tested on urine and saliva samples without any previous processing. Moreover, the comparison between their SPE model and an ELISA assay revealed many advantages offered by the SPE-based immunosensor, such as lower costs, high specificity, high sensitivity, fast results, and no need for sample processing before analysis. 

Reports of rapid and effective sensors to detect psychotropic drugs have also been declared. Scientists used NH2-graphene screen-printed electrodes modified by MIP technique with poly-pyrrole to detect methcathinone and cathinone [44]. Among the qualities exhibited by the sensor they developed were fast absorption, good selectivity, and exceptionally good reuse capacity.

A sensor used to detect cocaine in complex matrices (saliva, river water, and street samples) was also constructed via the MIP technique and it showed good selectivity and reproducibility. The sensor was fabricated using a graphene-modified screen-printed electrode, coated with palladium nanoparticles. They used p-aminobenzoic acid monomers and cocaine as template for the electro polymerization of the polymeric film onto the graphene-modified SPE. For the evaluation of the ability of the newly designed sensor to detect and quantify cocaine, square wave voltammetry (SWV) was used [45].

A summary of the SPE functionalization methods used to detect illicit drugs are presented in Table 2.

## 5. Screen-Printed Electrodes for Early Cancer Diagnostic

According to National Vital Statistics, between 2002 and 2006 cancer incidence in white people was 470.6, 311.1 in Asian people, and 493.6 in black people. The data calculated for 100,000 persons from each group. Lung, breast, and prostate cancer are the most common types of cancer in USA and have caused the death of 227,900 people in 2007; approximately 14.5 million Americans have been diagnosed in 2015, according to the data from the National Cancer Institute [46].

The chances of beating cancer are low, despite the last 20 years of scientific advancements that increased the lifespan of many cancer patients. In order to have a better chance of dealing with cancer, diagnosis has to come at an incipient stage [46]. The actual diagnostic methods include the Papanicolaou test for cervical cancer, mammography for breast cancer, PSA (prostate-specific antigen) level for prostate cancer as well as CTX-rays and MRI. Most of these technologies are expensive, not very specific, and do not always detect cancer in its early stages.

In the last few years, a new technology has improved improving the chances of an incipient cancer diagnostic and treatment. SPE can diagnose a variety of cancer-related pathologies, such as breast cancer, lung cancer, and other types of cancer.

All cancer cells synthesize specific biomarkers that enables us to differentiate them from normal cells. Special attention should be attributed to find sensitive and selective methods for their detection. Methods for detecting cancer biomarkers through SPE are presented in this review. Among the detected biomarkers are Mucin1, tumor markers such as CA 15-3, squamous cell carcinoma (SCCA), antigen fragment 21-1, carbohydrate Ag 125, and neuron specific enolase (NSE), as well as biomarkers for human papillomavirus (HPV) detection.

### 5.1. Aptasensors for Cancer Detection

There are a series of aptamer-based protocols used for to electrochemically detect DNA sequences, different proteins, or other molecules. Although the aptamer-based technology provides good results, it raises some issues related to the optimal quantity of aptamer immobilization and the conductivity of the electrode [1]. 

The utilization of SPEs in the construction of an electrochemical aptasensor can counteract the problems that may arise with aptasensors. In a 2016 study, Nawaz et al. [47] developed an aptasensor for breast cancer biomarker detection. To immobilize aptamers, they used a new method involving the modification of the surface of carbon nanotubes (CNTs), as well as the working electrode graphene of a SPCE, in order to perform voltammetry and impedimetric measurements to detect the breast cancer biomarker. These measurements were taken from SPCE obtained with a new printing system DEK 248 and by using the Gamry Reference 3000 potentiostat. To obtain SPE with a graphene working electrode and CNTs it is necessary to do a functionalization of the working surface, and then perform de immobilization of the breast cancer specific aptamers. This functionalization is required because the CNTs surface is hydrophobic and has a low conductivity; the functionalization allows the surface to use the electrochemical methods CV and EIS. 

After optimization, an aptasensor was used for the quantitative analysis of mucin. Results showed the directly proportional growth of the mucin with the increase of electron transfer resistance (R_et_), thus the interaction of the mucin with the aptamer caused an increase of the ∆ ratio. Nawaz et al. [47] also presented the high specificity of the diagnostic by using the screen-printed carbon electrode (SPCE) aptasensor, the interference of any other molecule with mucin being insignificant. The aptasensor was also verified with real human samples, the results showing the clear possibility of using this method to detect MUC1, having a good reproducibility and sensitivity [47].

The MUC1 gene encodes for mucin 1 protein and is an integrated part of the epithelial membrane of breast, bladder, pancreas, and ovary. It has a high importance within the membrane, the abnormal levels being immediately detected by using special aptasensors. Another example of a biosensor was presented by Florea et al. [48], with the immobilization of MUC1 on a graphite surface, respectively on a gold one within the SPE. The electrochemical EIS and CV techniques were used to test the aptamer-MUC1 complex and the results showed a higher detection limit on the graphite surface (3.6 ng/mL^−1^), compared to the gold surface (0.95 ng/mL^−1^).

### 5.2. Immunosensors for Cancer Detection

The biomedical field is extremely dynamic New products appear often, new improvements of already existing products are frequent, and devices for "in vitro" diagnosis are no exception.

Research in the field of immunosensors, through the appearance of miniaturized reaction surfaces, has revolutionized the medical industry. These new methods are multidisciplinary and include the latest results and technologies in the field of materials science, chemistry, biochemistry, molecular biology, information technology, electronics, microfluidics, and biophysics.

Globally, the use of immunosensors confers an important direction because of the benefits they offer as diagnostic devices. Immunosensors are easy to use, results are obtained in a short time, no sophisticated equipment or special working conditions are required, analytical performances are superior to current methods, and costs are low [49].

An interesting study based on the use of an immunosensor with anthraquinones (Aq) immobilized for HPV type 16 DNA detection has previously presented. The Dyµ ID was formed with a double-sided adhesive card and a SPE with eight working electrodes. The device consists of one counter electrode, a reference electrode and eight working electrodes. The antibody was added to the SPE array and this new compound was used to form the Dyµ ID system. Magnetic particles (MPs) modified with polyclonal antibodies were used to detect the biomarker. The detection limit obtained for the carbohydrate antigen 15-3 (CA15-3) was 6 micro units (µU/mL^−1^), requiring only 2 µl of serum for eight simultaneous detections. The results obtained by Oliveira et al. [50] confirmed the possibility of using immunosensors for breast cancer detection. The prototype was tested on samples from breast cancer patients and compared with the commercial chemiluminescence test currently used [50].

In another recent study, a group of researchers presented the deficiencies that occur when using SPE, as well as a method to improve the technology and eliminate the small problems that exist. One of these is the use of several working electrodes and a single counter-electrode, which leads to interference of current measurements within the counter-electrode due to its small surface area, affecting the repeatability of the analysis. Another problem is the use of expensive metals such as Ag or platinum, which increases the price of the method. Moreover, the sensitivity may be affected by the presence of binding polymers in the carbon ink used for printing SPE. In order to counteract these possible problems, SPE has been realized to attach high conductive materials to working electrodes, as well as to use one signal output channel and leave the reference electrode and counter electrode outside of SPE. Thus, the specificity could be increased by the distinct, individual modification of each working electrode so that each one could use the target of interest [51].

To test this new design of SPE, four tumor markers were immobilized, each on a working electrode: squamous cell carcinoma (SCCA), antigen fragment 21-1, carbohydrate Ag 125, and neuron specific enolase (NSE). This system with the counter and reference electrodes outside SPE was compared with ordinary SPEs and the results obtained using cyclic voltammetry (50 cycles) in 5 mM (Fe (CN)_6_) showed improved stability for the new design, as well as the lack of interference. This new SPE model was also tested on human serum samples and compared with a commercial ELISA method. The error between the two was about 8%, which shows that this immunosensor gives good detection results for tumor markers. Due to the high specificity and the possibility of modifying working electrodes for a particular target, the proposed SPE can be used to test any type of molecule [51].

### 5.3. Aptasensors versus Immunosensors for Cancer Detection

An interesting study was carried out by Sristava et al. [52], who compared the efficacy of aptasensors with immunosensors for the early detection of PSA. In general, antibodies have a low pharmaceutical value due to their size, which is why aptamers that have a 3D structure have better specificity and affinity. For this purpose, as well as due to the possibility of binding molecules with high affinity, aptamers have been used more in recent years as substitutes for antibodies (Abs). An essential feature of aptamers is their ability to be chemically modified, having many thiol groups, as well as many other functional groups [52].

The PSA immunosensor consists of SPE modified with graphene quantum dots-gold nanorods (GQDs-AuNRs). Commercial SPEs modified with a GQDs-AuNRs compound containing chitosan were used. The immobilization of Ab on the immunosensor in the aptamer PSA was performed immediately after. The testing and comparison of the two biosensors was done using cyclic voltammetry and EIS. The results suggest a higher applicability of the aptasensor compared to the immunosensor due to its superior stability, simplicity, and cost effectiveness [52]. Although both options of sensors were efficient, with similar detection limits (approximately 0.14 ng mL^−1^), the results obtained using aptasensors were slightly better than those using immunosensors in terms of accuracy and specificity [52].

A synthesis of all sensor variants for cancer-specific biomarkers is presented in Table 3, with details regarding the methods of functionalization, immobilization of the capture probe and electrochemical techniques used for their detection/quantification.

## 6. Metabolic Syndrome Specific Biomarker C-Reactive Protein (CRP) Detection Based on Screen-printed Electrodes

MS is defined as a conglomerate of metabolic changes, determined by the interaction of genetic, metabolic, and environmental factors, which can lead to a number of complications [53]. The syndrome name was proposed by WHO in 1998 and comes from the insulin resistance that accompanies the perivisceral deposition of adipose tissue (abdominal or centripetal obesity) that induces insulin resistance, increasing the risk of developing ischemic heart disease (following atherosclerosis (ATS)) and hypertension from other health problems, such as diabetes and stroke [54,55]. Insulin resistance is the main mediator in the installation and evolution of MS.

The prevalence of MS has increased worldwide in recent decades. MS is increasingly prevalent in developed and developing countries; in the US approximately 1/3 of adults have this syndrome, while in South Asia approximately 30% of adolescents suffer from this syndrome and their proportion is constantly increasing in developed countries [55,56]. For example, an increase in the number of people with diabetes is estimated at up to 592 million by 2035, with a large proportion of these individuals (up to 174.8 million) remaining undiagnosed. From a clinical point of view, it is important to identify people at risk of diabetes and metabolic disorders to prevent long-term complications and to reduce the costs of treating diabetes. The total costs, related to the treatment of diabetes, including the costs related to the prevention of complications and the economic losses (e.g., hospitalization, medical leave) are in the order of billions of euros, according to a study performed on patients from Germany, Spain, and Italy. [57].

In addition to the diagnostic criteria exposed by the WHO, there are serum markers that constitute "additional" criteria, which are important due to the complications they can cause, such as increased LDL-cholesterol (low-density lipoprotein) concentration, increased protein C level reagents, and increased fibrinogen concentration.

Recently, three biomarkers have been found to help diagnose MS: leptin/adiponectin ratio, adipocyte fatty acid binding protein (A-FABP), and C-reactive protein.

### Screen-Printed Electrodes for CRP Detection

CRP is a plasma protein with a pentamer structure and molecular mass of about 110–140 kDa. C-reactive protein always shows the inflammation in the body, being increasingly used as a biomarker for the assessment of many disorders or for the risk of developing disorders. It is used in autoimmune disorders like Crohn’s or Ulcerative Colitis to show that there is an inflammation in the body and that the disease is in the active phase. It is also used a cardiovascular risk and recently there have been many studies that show the importance of this protein in the diagnostic process of MS. C-reactive protein is produced by the body when the walls of blood vessels are inflamed. The higher CRP levels, the higher the inflammation level of the body. This protein plays an essential role in the evolution of chronic inflammation, being the result of constant damage to the internal walls of the arteries. These transformations are the result of an unhealthy lifestyle, especially with food choices that lead to excess LDL-cholesterol, triglycerides, and blood glucose, as well as arterial hypertension (AH). CRP protein is predominantly produced by the liver and is strongly associated with MS [53,58]. Increasing the level of C-reactive protein (acute inflammation test) is an indicator of the general pro-inflammatory status of the body, which suggests an increased risk of cardiovascular disease. The explanation for the increase of this acute phase inflammation protein is related to the presence of fat stored abdominally, adipocytes releasing circulating cytokines/adipokines with proinflammatory role, with increased risk of cardiovascular disease [59].

The current methods for determining C-reactive protein are ELISA (which requires a long analysis time) and Latex. These methods, however, have a relatively low sensitivity and repeatability, as well as a higher price, which is why the use of rapid sensor-based tests to determine CRP level is becoming more up-to-date [60]. Such a model was proposed by Kumar and Prasad [61] who used molecularly imprinted polymer (MIP) technology on a modified SPCE for quantitative determination of CRP in human serum. They started to obtain the new diagnostic method by treating the screen-printed electrodes using electrochemical methods to raise their sensitivity. After that they performed MIP grafting onto the SPCE surface. The measurements were taken with DPV and cyclic voltammetry (CV) recorded on a portable potentiostat-Stat 200 from Drop Sens. The new SPE immunosensor was tested with human serum. The results were accurate without interference and without false-positive results.

A different approach of designing SPE for CRP determination is the use of the origami paper-based immunoassay. This type of method was presented Boonkaew et al. [60] and started with the modification of a graphite screen-printed electrode to raise sensitivity. The method used gold nanoparticles electrodeposited on the surface of the SPCE and L-cysteine as a self-assembled monolayer. Moreover, hexacyanoferrate was used a as a redox probe in order to check the charge-transfer resistance of the electrode. Usual electrochemical measurement CV and scanning electrochemical microscopy (SEM) were used to test the new SPCE. The detection limit found was 15 ng/mL^−1^ and the efficiency of the technique was confirmed by the CRP determination on human serum samples [61].

**Figure 4:**
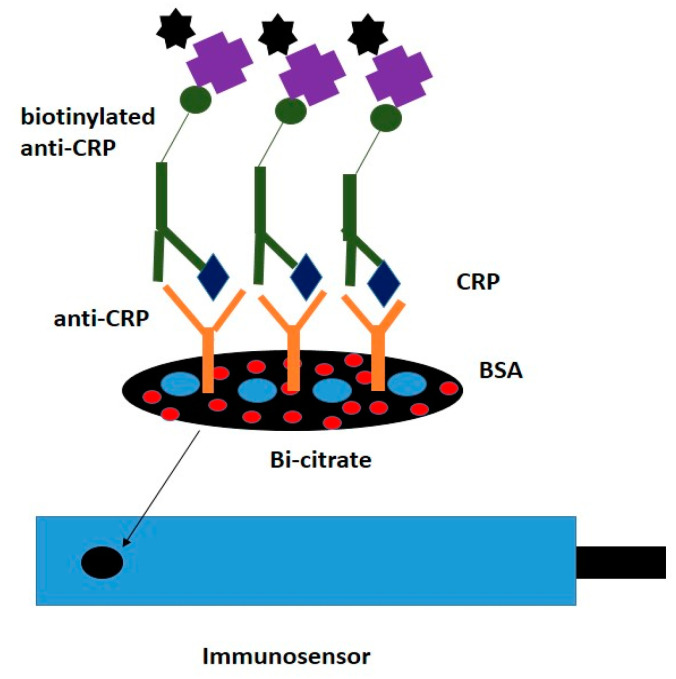
Sandwich-type immunoassay on a modified SPE [62].

A new immunosensor model was presented by Kokkinos et al. [62] based on the use of SPE graphite to which bismuth citrate was added (Figure 4), and the detection of C-reactive protein from human serum samples was performed using cyclic voltammetry. After immobilizing Ab anti-CRP on the surface of a SPCE manufactured on a polyester paper, voltammetry tests were performed using a reference electrode based on Ag/AgCl and a Pt wire counter electrode [62].

In the assay Streptavidin-conjugated, Pbs QDs were used to aid in protein determination. The results showed a CRP detection limit of 0.05 ng·mL^−1^. The values were similar, between 0.2–100 ng/mL^−1^ CRP, which showed that the method had great repeatability and specificity [61].

Carbon screen-printed electrodes have been modified through a series of methods using various compounds in order to obtain cost-effective and high-performance sensors for quantitative determination of CRP in human serum. The details regarding the way each biosensor was constructed are presented in Table 4. 

## 7. Evaluation of the Performance of SPE-Based Sensors versus Other Diagnosis Methods

The standard sandwich ELISA immunoassay determines a color change in the substrate, which translates in a detectable signal that increases in a linear fashion in time. This aspect makes ELISA-based diagnosis methods have limited sensitivity, as opposed to most SPE-based methods, which were shown to have better performance especially when carbon nanotubes and sodium alginate and carboxymethyl chitosan were used for their functionalization [63].

A series of methods were used to detect various diseases or disease inducing microorganisms, such as molecular analysis (PCR, real-time PCR, LAMP, etc.) and immunological methods (ELISA or lateral flow immunoassay). However, each method seems to have its limitations in regards to specificity, sensitivity, selectivity, or detection limit [64]. Although molecular diagnosis methods are superior in terms of performance, they involve sample processing, higher costs, and are often time consuming as opposed to electrochemical diagnosis methods using SPE.

Some studies have reported a detection limit for pathogenic microorganisms as low as 68 CFU mL^−1^ in PBS and 6.8 × 10^2^ to 6.8 × 10^3^ CFU mL^−1^ in food matrix when using an improved ELISA [65]. Studies carried out using modified SPEs seem to go as low as 400 cells/mL to 800 cells/mL. According to the data provided by other researchers, the detection limit of a typical ELISA for the determination of a protein target varies widely, between 0.01 pg mL^−1^ to 100 ng mL^−1^. When evaluating the performances of SPE-based sensors to detect protein targets, we noticed that the detection limit usually varies between 0.05 ng mL^−1^–15 ng/mL^−1^ [66].

Although SPEs are important candidates for rapid diagnosis, they have limitations. For instance, cross-reaction with other non-target products can occur, which leads to poor selectivity of the method [10]. As observed by us in practice, commercially available SPEs appear to have a thin layer of silver on the reference electrode. Thus, after a few cycles of CV and impedance measurements, it tends to significantly degrade. Moreover, after functionalization, the storing conditions must be rigorously controlled because their stability is poor even in refrigerated conditions. For these reasons, we must consider the fact that besides the performance of a SPE-based biosensor we must also pay attention to the conservation of this type of sensor.

Some scientists found a way to merge the ELISA principle with SPEs in order to construct a hybrid microfluidic sensor capable of detect traces of cocaine in water and body fluids [67]. New and improved diagnosis methods can also be obtained by combining molecular techniques for the amplification of specific sequences and functionalized screen-printed electrodes to detect the amplified product.

## 8. Conclusions

Screen-printed carbon electrodes seem to be the favorite choice of researchers when it comes to developing fast and cost-effective methods to detect/quantify disease inducing agents. However, the modification of these electrodes with gold nanoparticles or gold thin films is often required in order to increase the electrochemical signal and/or the immobilization surface. Another fact worth mentioning is that aptamer-based sensors showed a slightly better specificity and affinity for cancer related biomarkers in comparison to antibodies-based sensors (immunosensors). It is obvious that no matter the model of biosensor constructed using various materials and functionalization patterns, screen-printed electrode-based biosensors represent a serious candidate in the development of rapid in vitro diagnosis methods with a variety of applications. This could lead to important progresses in the early diagnosis of life-threatening diseases, as well as epidemiological control of infectious diseases and public health assurance by increased control of food products and drug trafficking and abuse.

## Figures and Tables

**Table 1:** Functionalization of various screen-printed electrodes (SPEs) to detect pathogens.

SPE Type	Activation/Functionalization Patterns	Compounds	Immobilization	Electrochemical Methods	Reference
**Screen-Printed Carbon Electrode**	Surface modified using multiwalled carbon nanotubes	Carbon nanotube-polyallylamine	NC ^1^-antibodies conjugates (metal-based nanocrystals)	Square-wave voltammetry	[9]
	Sandwich-like immunoassay	Reduced graphene oxide gold nanoparticles silica immunomagnetic beads	Ab1 conjugation to silica beads Ab2 linked to rGO ^2^/AuNPs ^3^	Differential pulse voltammetry	[11]
	Surface modified by composite films	Multi-walled carbon nanotubes (MWCNTs)/sodium alginate (SA)/carboxymethyl chitosan (CMC) composite	HRP ^4^-labeled antibodies immobilized on MWCNTs/SA/CMC complex	Cyclic voltammetry	[14]
	Surface modified with gold nanoparticles using glutaraldehyde as cross-linker	Glutaraldehyde Gold nanoparticles 1,1′-ferrocene-dicarboxylic acid (FeDC) (mediator)	Immobilization of Ab on the gold nanoparticles	Cyclic voltammetry and amperometry measurements	[15]
	Surface modified with agarose-Nano-Au membrane	Gold nanoparticles Agarose solution	HRP-labeled antibody immobilized on agarose-Nano-Au membrane	Cyclic voltammetry	[20]
	Gold modified SPCE ^5^ for hybridization-based genosensor	Gold nanoparticles Synthetic oligonucleotides	Oligonucleotide probes were fixed on the Au-NP through a thiol group attached to the 3′-end	Cyclic voltammetry	[25]
	Sandwich-like immunoassay	Tosyl-activated Magnetic Beads Gold nanoparticles	Magnetic Beads coated with target antigen, followed by binding of Ab1	Chronoamperometry	[28]
	Nanocomposite modified surface	Nafion/TiO_2_ rhodium nanoparticles	Ab ^6^ immobilization on rhodium nanoparticles	Differential pulse voltammetry EIS ^7^	[29]
	Carbon ink and thiophene on a polyethylene terephthalate and AuNP-Ptn A	Thiophene Protein A Gold nanoparticles	Ab immobilization on the gold nanoparticles	Cyclic voltammetry	[30]
	Sandwich-like immunoassay using home-made SPE made from silver and carbon ink on a polystyrene substrate	Polystyrene Mesitylene graphite particles	Incubation of home-made immunosensor with capturing antibody for 1 h (physical adsorption)	Cyclic voltammetry	[31]
	Surface coated with agarose/Nano-Au membrane and horseradish peroxidase (HRP) labeled antibody (HRP-antibody)	Agarose HRP-antibody Nano-Au	HRP-antibody Agarose-Nano-Au immobilized on polyethylene	Cyclic voltammetry	[32]
**Screen-Printed Gold Electrode**	Amine coupling of carboxymethyl dextran to the gold surface	Carboxymethyl dextran N-ethyl-N′-(3-dimethylaminopropyl)-carbodiimide (EDC) N-hydroxysuccinimide (NHS)	Binding of the Ab to carboxylic groups of carboxymethyl dextran	Chronoamperometry	[10]
	3,3 dithiodipropionic acid di(*N*succinimidyl ester) (DTSP)-based self-assembled monolayers (SAMs)	DTSP	Ab binding through primary amino groups to ester groups of DTSP Or by Thiolated antibodies	Amperometric measurements	[12]
	Genosensor obtained by immobilization of thiolated capture sequence on thin-film gold electrodes	Thiolated capture synthetic oligonucleotides	Thiol-functionalized oligonucleotide probes were bind via gold-sulfur interaction	Differential pulse voltammetry	[13]
	Surface functionalization with biotinylated bovine serum albumin (BSA) solution, streptavidin, and nano-Yeast scFv	Biotinylated BSA streptavidin	Ab binding via biotin-streptavidin complex	Differential pulse voltammetry	[34]
	Surface modification with polymers-polytyramine (Ptyr)	Polytyramine NeutrAvidin	Ab binding via biotin-NeutrAvidin coupling	Cyclic voltammetry Electrochemical Impedance Spectroscopy	[35]

^1^ nanocrystals; ^2^ graphene oxide; ^3^ gold nanoparticles; ^4^ horseradish peroxidase; ^5^ screen-printed carbon electrode; ^6^ antibody; ^7^ electrochemical impedance spectroscopy.

**Table 2:** Functionalization of various SPEs to detect illicit drugs.

SPE Type	Activation/Functionalization Patterns	Compounds	Immobilization	Electrochemical Methods	Reference
**Screen-Printed Carbon Electrode**	Functionalization via molecularly imprinted polymer-4-aminobenzoic acid and MWCNT	4-aminobenzoic monomers MWCNT Naloxone (template)	Electropolymerization of the polymeric film on the surface of the electrode modified with MWCNT	Differential pulse voltammetry	[39]
	Surface modification by mediators that once oxidized react with drugs such as MAMP ^1^ or Δ9-THC ^2^.	N-(4-amino-3-methoxyphenyl)-methanesulfonamide) N,N′-(1,4-phenylene)-dibenzenesulfonamide	NA	Cyclic voltammetry	[41,42]
	Direct immobilization of Ab on SPCE by passive absorbtion	Antibodies HRP marked target sample	Passive absorbtion of Ab under humidity conditions	Amperometric measurements	[43]
	Deposition of molecularly imprinted polymer–polypirrole on SPE modified by NH2-graphene	Pyrrole monomer target drug templates (methcathinone and cathinone)	Electropolymerization of the polymeric film on the surface of the electrode modified with NH2-graphene	Cyclic voltammetry Electrochemical impedance spectroscopy	[44]

^1^ methamphetamine; ^2^ delta-9-tetrahydrocannabinol.

**Table 3:** Functionalization of various SPEs used to detect cancer related biomarkers.

Sensors Type	Activation/Functionalization Patterns	Compounds	Immobilization	Electrochemical Analysis Technics	Reference
**Aptasensors**	Covalently functionalized CNTs ^1^ on screen-printed carbon electrodes in the construction of an electrochemical aptasensor	MWCNTs, orthodichlorobenzen, 4-aminobenzoicacid, acetonitrile	The terminal benzoic acid groups on SPCE surface were activated by immersing the SPCE into a solution of 100 mM N-(3-dimethylaminopropyl)-N0-ethylcarbodiimide hydrochloride (EDC) Aptamer solution was incubated onto the activated SPCE.	Electrochemical impedance spectroscopy	[47]
	The surface of working electrode was modified with nanoparticle (AuNPs) by electrodeposition from solution.	A solution of HAuCl_4_ 0.6 M in H_2_SO_4_ 0.5 M Electrodeposition of gold on SPEs, after electrochemical cleaning with 0.5 M H_2_SO_4_ by potential scanning within a range of −0.2 to 1.2 V		Electrochemical impedance spectroscopy Differential pulse voltammetry Cyclic voltammetry	[48]
	WE surface was modified by graphene quantum dots and gold nanorod on a thin film of chitosan.	Chitosan Graphite powder H_2_SO_4_ HNO_3_ N-cetyl-N, N, N-Trimethyl Ammonium Bromide (CTAB) HAuCl_4_ NaBH_4_	Aptamer solution was added to the WE and then it was stored overnight in a humid chamber	Cyclic voltammetry Electrochemical impedance spectroscopy Differential pulse voltammetry	[52]
**Immunosensors**	Microfluidic immunoarray device (Dyμ ID) was based on the use of a double-sided adhesive polystyrene card with the microfluidic channel used for sealing the device and a screen-printed array with eight electrodes as working electrodes, one counter electrode, and one reference electrode The immunoarray was modified using the layer-by-layer technique aiming at immobilizing the primary antibody	The volume of the magnetic particles modified with the polyclonal antibody and horseradish peroxidase (MP ^2^-Ab2-HRPs) added in the capturing step and incubation and capture time along with the flow rate	Polyclonal antibodies were bound to (MPs) and peroxidase enzymes were used as a strategy for capture, separation, and preconcentration of the biomarker, in addition to amplification of the electroanalytical signal	Cyclic voltammetry Electrochemical impedance spectroscopy	[50]
	Four different solutions were prepared for the individual functionalization of each WE ^3^: poly (o-phemylenediamine)-Au/Pd (PoPD-Au/Pd), poly (methylene blue)-Au/Pd (PMB-Au/Pd), poly (N, N′-diphenyl-p-phenylediamine)-Au/Pd (PPPD-Au/Pd) and poly (3, 3′, 5, 5′-tetramethylbenzidine)-Au/Pd (PTMB-Au/Pd). After preparation each solution was mixed with sodium alginate and gold nanoparticles, and then 10 μL of each solution was added to each of the 4 WE	Poly (o-phemylenediamine) Poly (methylene blue) Poly (N, N′-diphenyl-p-phenylediamine) Poly (3, 3′, 5, 5′-tetramethylbenzidine)	Antibody solutions were added on each WE and incubated for 12 h	Square wave voltammetry	[51]
	WE surface was modified by graphene quantum dots and gold nanorod on a thin film of chitosan.	Chitosan Graphite powderH_2_SO_4_ HNO_3_ N-cetyl-N, N, N-Trimethyl Ammonium Bromide (CTAB) HAuCl_4_ NaBH_4_	Antibody solution was added to the WE and then it was stored overnight in a humid chamber.	Cyclic voltammetry Electrochemical impedance spectroscopy Differential pulse voltammetry	[52]

^1^ carbon nanotubes; ^2^ magnetic particles; ^3^ working electrode.

**Table 4:** Different designs of SPEs to detect CRP.

Electrode Type	Activation/Functionalization Patterns	Compounds	Immobilization	Electrochemical Analysis Technics	Reference
**Carbon Screen-Printed Electrode**	First of all, the WE was modified with graphene to enhance sensitivity. Then, gold nanoparticles were electrodeposited on the modified surface and self-assembled monolayer of L-cysteine	L-cysteine Graphene powder Potassium tetrachloroaurate (III)	The specific antibody was covalently immobilized, after the activation of the carboxyl groups with EDC/NHS solution	Electrochemical impedance spectroscopy	[60]
	Surface modification through MIP technique using 2-Acryl amidoethyldihydrogen phosphate (AEDP) and N-(4-dimethylaminophenyl)-acrylamide (DMAA). The sensitivity of the designed model was enhanced by the addition of multiwalled carbon nanotubes (MWCNTs)	2-Acryl amidoethyldihydrogen phosphate N-(4-dimethylaminophenyl)-acrylamide MWCNTs	No capture molecule was required	Cyclic voltammetry Differential pulse voltammetry	[61]
	Surface modification with Bismuth Citrate for the development of a sandwich-type assay	Graphene Bismuth citrate Bovine serum albumin	Capture antibody was immobilized by physical absorption onto the surface of the WE. The second biotinylated Ab tied to streptavidin-conjugated quantum dots.	Anodic stripping voltammetry (ASV)	[62]
